# Magnetic Clustering Effect during the Association of Biofunctionalized Magnetic Nanoparticles with Biomarkers

**DOI:** 10.1371/journal.pone.0135290

**Published:** 2015-08-13

**Authors:** Kuen-Lin Chen, Jean-Hong Chen, Su-Hsien Liao, Jen-Je Chieh, Herng-Er Horng, Li-Min Wang, Hong-Chang Yang

**Affiliations:** 1 Department of Physics, National Chung Hsing University, Taichung, Taiwan; 2 Department of Materials Engineering, Kun Shan University, Tainan, Taiwan; 3 Institute of Electro-Optical Science and Technology, National Taiwan Normal University, Taipei, Taiwan; 4 Graduate Institute of Applied Physics and Department of Physics, National Taiwan University, Taipei, Taiwan; 5 Department of Electro-optical Engineering, Kun Shan University, Tainan, Taiwan; RMIT University, AUSTRALIA

## Abstract

We report herein an investigation into dynamic magnetic clustering that occurs during immunoassays as biofunctionalized magnetic nanoparticles (BMNs) become associated with biotargets. We measure the dynamic effective relaxation time *τ*_eff_(*t*) and use scanning electron microscopy (SEM) and transmission electron microscopy (TEM) to investigate the C-reactive protein (CRP) as it associates with the BMN Fe_3_O_4_-antiCRP to form the magnetic cluster Fe_3_O_4_-antiCRP-CRP. The results indicate that *τ*_eff_(*t*) increases with increasing association time. In addition, the ration Δ*τ*_eff_/*τ*_0_ as a function of CRP concentration follows a characteristic logistic function, which provides a basis for estimating the quantity of biomolecules with a detection sensitivity close to 0.1 ppm. After the association, SEM and TEM images show that CRP and Fe_3_O_4_-antiCRP conjugate to form Fe_3_O_4_-antiCRP-CRP clusters hundreds of nanometers in size. The SEM and TEM images provide direct evidence of the formation of magnetic clustering.

## Introduction

Magnetic detection using biofunctionalized magnetic nanoparticles (BMNs) has recently received considerable attention [1]. In general, biological samples have negligible magnetic backgrounds, therefore a high detection sensitivity and specificity is possible with magnetic detection. Magnetic detection for assaying biomolecules can be done by measuring the magnetic relaxation [2,3], magnetic remanence [4], immunomagnetic reduction (IMR) [5], saturated magnetization [6], reduction in spin-spin relaxation time [7], etc. In immunomagnetic assays, the relaxation in the presence of magnetic clustering during association plays an important role in the detection of biomolecules [1–3,5,7]. Because relaxation mechanism dominates the magnetic variance of sample, it is also the key point of sensitivity limit of the immunomagnetic assay. However, the effects of dynamic magnetic clustering on the characteristics of relaxation are seldom studied. A previous report [8] discusses the molecule-assisted nanoparticle clustering effect in immunomagnetic reduction (IMR) assay. In that study, clustering effects were manipulated by controlling the concentrations of anti-body-functionalized magnetic nanoparticles in the reagent. Mixed-frequency IMR was used to study the clustering effect, and the experimental results show that particle clustering is enhanced by increasing the concentration of BMNs. In addition, this report claims that the effects of magnetic clustering dominate the increased rate of the IMR signal in mixed-frequency ac susceptibility. It clearly showed that the variance of ac susceptibility due to magnetic clustering effect is a promising method for biomolecule detection. Another report [9] shows real-time Brownian relaxation of BMNs by using a mixed frequency method. Due to clustering effects, the phase delay *θ*(*t*) between magnetization *M*(*t*) and applied field *H*(*t*) was varied during association. The dynamic behavior of the phase delay angle was studied for assay biomolecules. However, there are few reports on the direct observation of magnetic clustering. Liao et al. [10] reported the time-dependent phase lag of BMNs conjugated with biotargets, as studied with ac magnetic susceptometry for liquid phase immunoassays. Because of the clustering effect, Brownian relaxation of BMNs is suppressed, which in turn enhances the effective relaxation time. By monitoring the dynamic phase lag, a sensitive platform for assaying human C-reactive protein (CRP) was demonstrated. The research goal of the present work is to study the effects of magnetic clustering on the dynamic effective relaxation time *τ*_eff_(*t*) and provide evidence of magnetic clustering by imaging with scanning electron microscopy (SEM) and transmission electron microscopy (TEM).

In the present work, we report how dynamic magnetic clustering affects *τ*_eff_(*t*) by measuring the time-dependent phase lag *θ*(*t*) between the applied field *H*(*t*) and magnetization *M*(*t*) and by imaging with SEM and TEM. The measured phase lag *θ* is used to estimate *τ*_eff_ by using the relation tan*θ*(*t*) = *ωτ*_eff_(*t*), where *θ*(*t*) is the time-dependent phase lag of *M(t*) with respect to *H*(*t*), and *ω* is the angular frequency of the excitation. The BMNs used in this study were anti-goat C-reactive protein coated onto dextran-coated magnetic nanoparticles (Fe_3_O_4_-antiCRP), and the biotargets were human C-reactive protein (CRP). The results show that *τ*_eff_ for the reagents increases as association evolves, which we attribute to suppressed Brownian motion due to the presence of magnetic clusters during the association of CRP with Fe_3_O_4_-antiCRP. We also find that the ratio Δ*τ*_eff_/*τ*_0_, as a function of CRP concentration follows a universal characteristic logistic function, where Δ*τ*_eff_ = [*τ*_eff_ (*t* = 0) − *τ*_eff_ (*t* = ∞)]. Finally, direct evidence of magnetic clustering is provided by SEM and TEM images, which show that the average particle size is about 67.5 ± 11.5 nm for Fe_3_O_4_-antiCRP, whereas the conjugated clusters of Fe_3_O_4_-antiCRP-CRP are about 250 nm in size.

## Materials and Methods

### Materials

**Fig 1:**
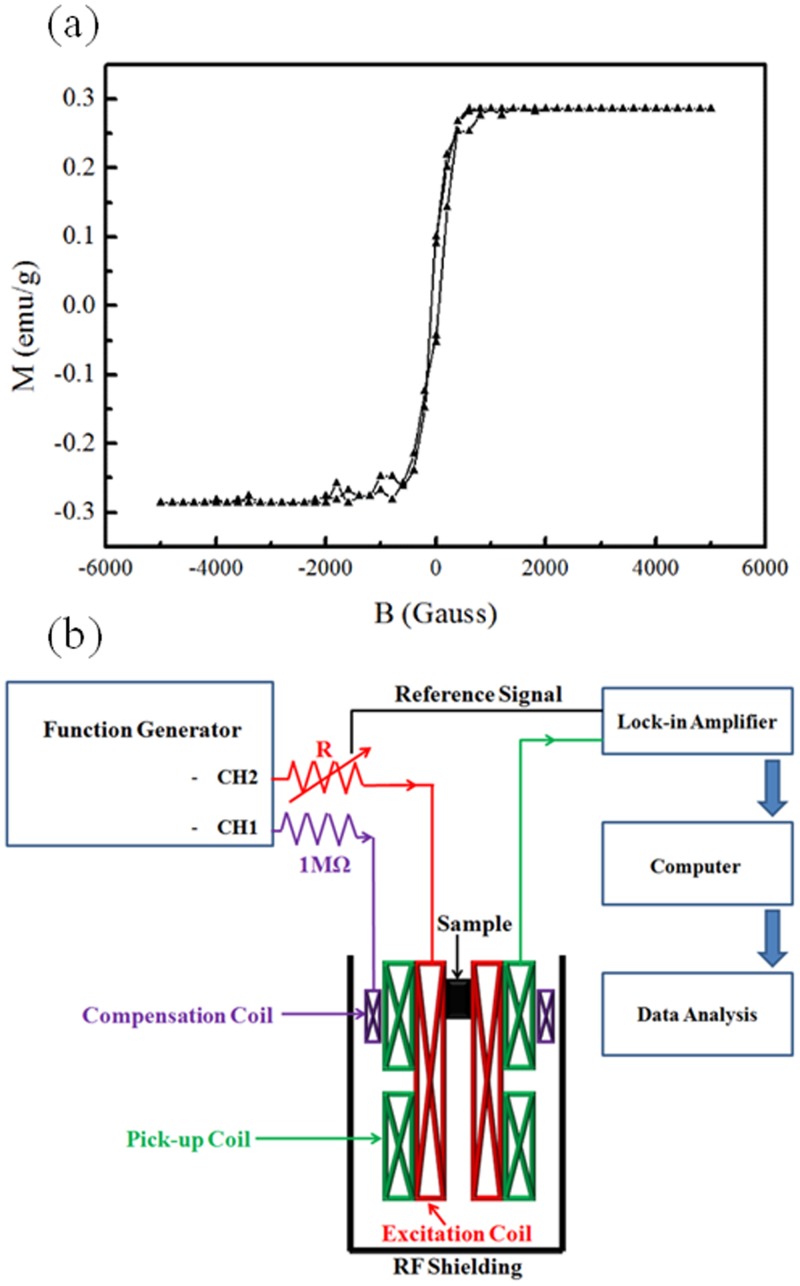
Detection scheme for homemade ac magnetic susceptometer for measuring dynamic *τ*_eff_(*t*).

The magnetic reagents used in this study were composed of biocompatible magnetic Fe_3_O_4_ nanoparticles with dextran shell (MagQu Co, Ltd). To synthesize BMNs, polyclonal goat anti-CRP (Sigma-Aldrich, St Louis, MO, USA) was covalently bound to the dextran shell of Fe_3_O_4_ via −CH = N− [11, 12]. BMN, which consisted of Fe_3_O_4_-antiCRP, was then dispersed in phosphate buffered saline (PBS) solution with a pH of 7.4. In this study, the mean hydrodynamic diameter of Fe_3_O_4_ nanoparticle was 45.8 nm with a standard deviation of ±15.0 nm, as determined by dynamic light scattering (Nanotrac-150, Microtrac). Fig 1a shows the magnetization data of the BMN reagent, which was measured by vibrating sample magnetometer (Model 4500, EG&G), and the saturated magnetization was about 0.29 emu/g.

### Magnetic ac susceptibility measurement

Fig 1b shows the design of the highly balanced homemade ac magnetic susceptometer used to study the time-dependent phase lag angle *θ*(*t*). A detailed description of the measurement technique for characterizing *θ*(*t*) is given in Ref. [10], so we only briefly describe the method here. The sensing unit of the ac susceptometer consists of an input coil, a first-order gradient pick-up coil, and a compensation coil. The first-order gradient pick-up coil is composed of two identical coils wound in opposite directions. The compensation coil is used to precisely balance the sensing unit in the ac susceptometer. Therefore, their phase difference can be adjusted to optimize the balance of the sensing unit. The balance of the sensing coil is defined as the output signal of the two coils of the pick-up coil divided by the output signal from one of the two coils under the excitation of the input coil. This sensing unit achieves a balance of 30 parts per trillion (ppt). The signal from the pick-up coil and the reference frequency component from the function generator were input to a lock-in amplifier, and the time-dependent *τ*_eff_(*t*) was estimated by using tan*θ*(*t*) = *ωτ*_eff_(*t*).

### SEM and TEM images

The morphologies of Fe_3_O_4_, Fe_3_O_4_-antiCRP nanoparticles, and CRP conjugated Fe_3_O_4_-antiCRP were investigated with field-emission scanning electron microscope (FESEM) (Hitachi S-4700), and field emission gun transmission electron microscope (FEI-TEM) (Philips Tecnai F20 G2). Before taking the electron microscopy, the nanoparticles and magnetic clusters were dispersed in a super-diluted solvent (1:50) and then drying the magnetic samples under vacuum at room temperature for 48 hours.

## Results and Discussion

**Fig 2:**
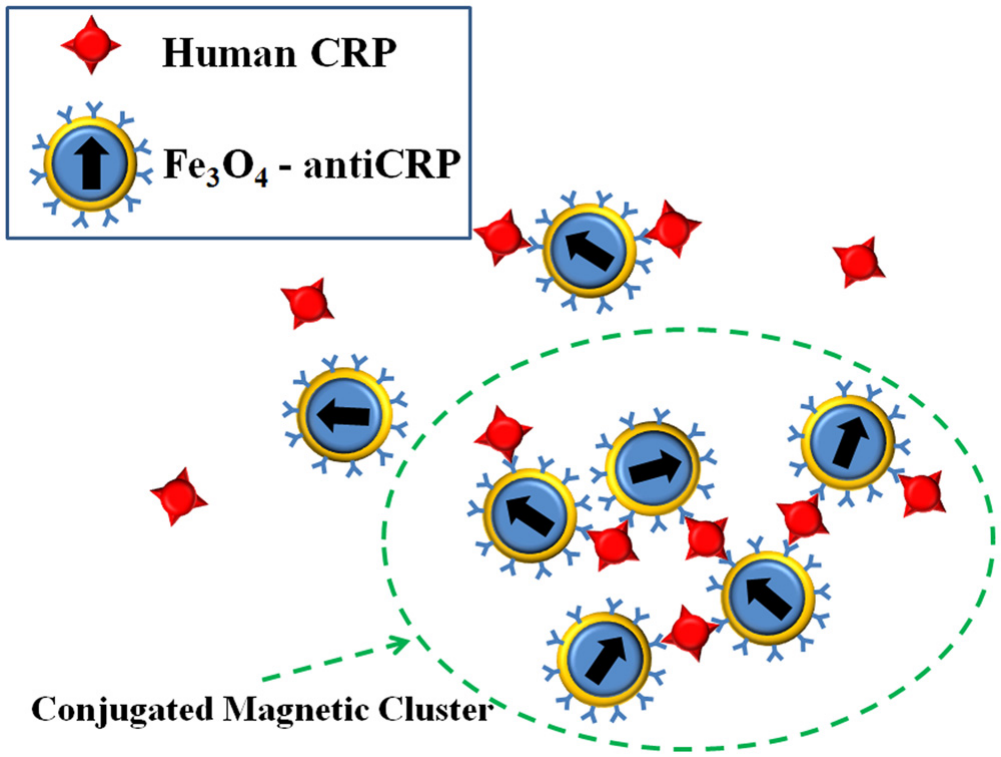
Illustration depicting conjugation of human CRP and Fe_3_O_4_-antiCRP to form Fe_3_O_4_-antiCRP-CRP cluster.

By means of the surface modification for specific biotargets, BMN is a good reagent for the immunomagnetic assay. By means of antigen-antibody interaction, BMNs will be gathered to form bigger clusters when the BMN reagent is mixed with antigen. Therefore, the magnetic characteristics of BMN reagent are going to change, and the variation of magnetic characteristics could be used to identify the quantity of antigen. An illustration depicting the human CRP, Fe_3_O_4_-antiCRP, and magnetic clusters consisting of Fe_3_O_4_-antiCRP-CRP is shown in Fig 2. In this work, we used magnetic ac susceptibility measurement to investigate the clustering effect of antiCRP coated BMNs interacting with CRP antigen. To study the phase lag dynamics *θ*(*t*), 60 μl of Fe_3_O_4_-antiCRP reagent of which saturated magnetization is 0.29 emu/g was mixed with 40 μl of CRP with concentrations varying from 0.1 to 10 ppm (1 ppm = 1 μg/mL). *θ*(*t*) is also related to *τ*_eff_(*t*) by tan*θ*(*t*) = *ωτ*_eff_(*t*), where *f* = *ω*/(2π) = 9 kHz is the excitation frequency used in the experiment. The presence of dynamic magnetic clustering during the association affects *θ*(*t*), which in turn modifies *τ*_eff_(*t*). We investigated the dynamics of *θ*(*t*) and *τ*_eff_(*t*) via lock-in detection, as shown in Fig 1b. The results show that *τ*_eff_ increases and saturates when association is complete. Furthermore, the ratio Δ*τ*_eff_/*τ*_0_ as a function of CRP concentration provides a basis for estimating the quantity of biomolecules. Finally, we directly observed the formation of magnetic clusters by SEM and TEM images.

**Fig 3:**
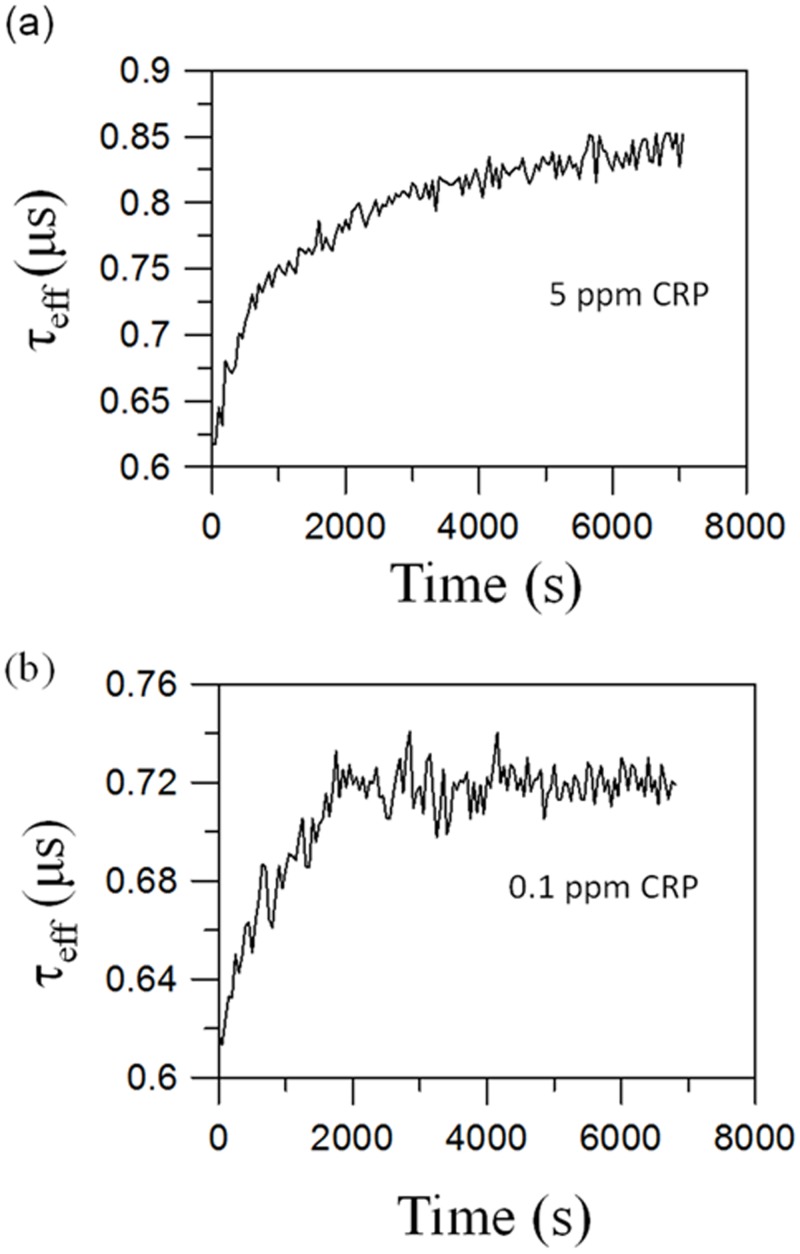
Time-dependent *τ*_eff_(*t*) for assaying (a) 5.0 ppm and (b) 0.1 ppm of human CRP. Upon conjugating Fe_3_O_4_-antiCRP and CRP antigen, *τ*_eff_(*t*) becomes larger and gradually approaches equilibrium.

Fig 3a shows the time-dependent *τ*_eff_(*t*) after mixing the reagent (0.29 emu/g) of Fe_3_O_4_-antiCRP with 5 ppm CRP at 300 K. The results show that *τ*_eff_ increases from *τ*_eff_ = 0.62 μs at t = 0 to *τ*_eff_ = 0.83 μs at t = 4800 s. Fig 3b shows *τ*_eff_ as a function of time after mixing Fe_3_O_4_-antiCRP with 0.1 ppm CRP at 300 K. The results show that *τ*_eff_ increases from 0.62 μs at t = 0 to *τ*_eff_ = 0.72 μs at t = 1800 s. The increase of *τ*_eff_ is attributed to the formation of magnetic clusters during the association, which depresses the Brownian relaxation. Completing the association of 5 ppm CRP with the reagents takes 1.5 hours. The association time decreases to 0.5 hours to assay 0.1 ppm CRP. Consequently, a shorter association time results from reducing the CRP concentration. Thus, characterizing the dynamics of *τ*_eff_ is done with a detection sensitivity of 0.1 ppm.

The mechanism behind the dynamics of *τ*_eff_ for BMNs during association can be understood as follows: Consider the ac magnetic susceptibility of BMNs, *χ*(*ω*), in an applied ac magnetic field. *χ*_ac_(*ω*,*t*) is a function of *τ*_eff_ and has angular frequency *ω*. The magnetic susceptibility *χ*_ac_(*ω*,*t*) can be expressed as [13,14]

$$\chi_{\text{ac}}(\omega ,t)=\chi \prime +i\chi \prime \prime$$ (1)

$$=\chi_{0}{\left\{{\left[\frac{1}{1+{\left(\omega \tau_{\text{eff}}\right)}^{2}}\right]}^{2}+{\left[\frac{\left(\omega \tau_{\text{eff}}\right)}{1+{\left(\omega \tau_{\text{eff}}\right)}^{2}}\right]}^{2}\right\}}^{1/2}{\text{e}}^{-i\theta (t)},$$ (2)

where *χ*_0_ is the component of *χ*_ac_ at *ω* = 0, *i* = (−1)^1/2^, *χ*' = *χ*_0_{1/[1+(*ωτ*_eff_)^2^]}, *χ*'' = *χ*_0_{(*ωτ*_eff_)/[1+(*ωτ*_eff_)^2^]}, and *θ* is the phase lag. *χ*''*(t*)/*χ*'(*t*) = tan*θ*, so Eqs 1 and 2 lead to tan*θ* = *ωτ*_eff_. For BMNs, *τ*_eff_ can be written as

$$1/\tau_{\text{eff}}=\sum_{\text{i}}1/\tau_{\text{i,B}}+\sum_{\text{i}}1/\tau_{\text{i,N}}=1/\tau_{\text{B}}+1/\tau_{\text{N}},$$ (3)

where Σ_i_ indicates summation over all BMN sizes, 1/*τ*_i,B_ is the relaxation rate due to the Brownian motion of nanoparticle i, and 1/*τ*_i,N_ is the relaxation rate due to the Néel relaxation of nanoparticle i. Using tan*θ*(*t*) = *ωτ*_eff_(*t*), the dynamics of *τ*_eff_ can be understood by monitoring the time evolution of *θ*. As Fe_3_O_4_-antiCRP conjugates with CRP, magnetic clusters form, which depresses the Brownian motion and in turn increases *τ*_eff_.

**Fig 4:**
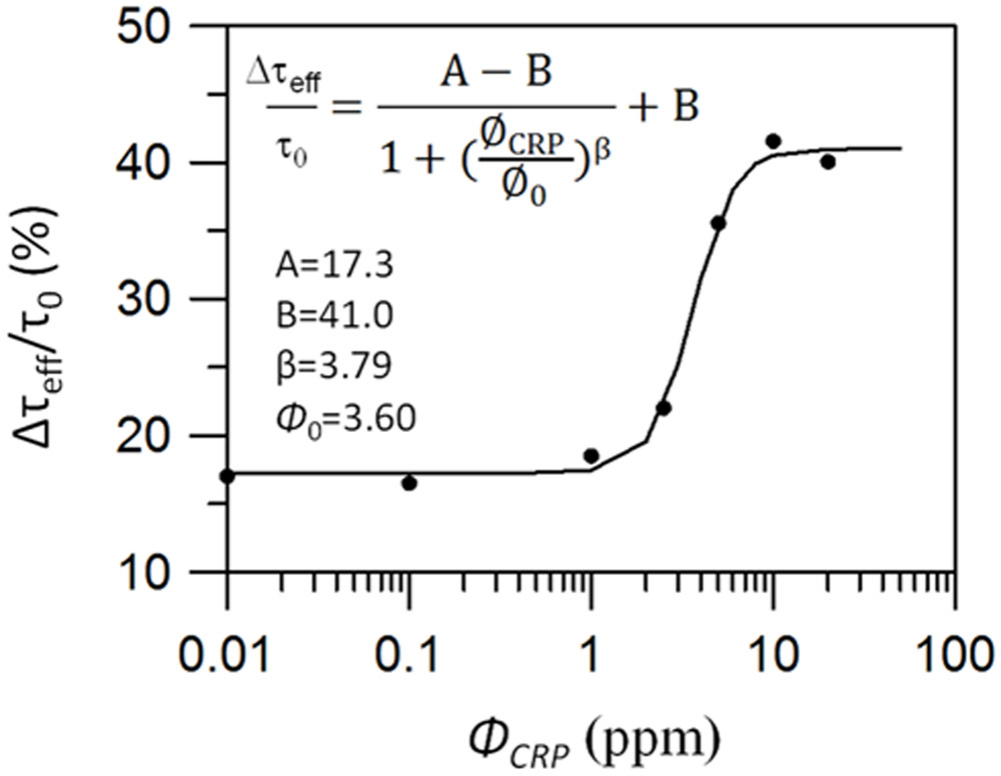
Δ*τ*_eff/_ *τ*_0_ as a function of CRP concentration in semilog plot at 300 K. *τ*_0_ is the effective relaxation time at t = 0. Δ*τ*_eff_ is the effective relaxation time between the beginning and the end of the reaction, and is defined as Δ*τ*_eff_ = [*τ*_eff_ (*t* = 0) − *τ*_eff_ (*t* = 6000 s)].

Fig 4 shows the ratio Δ*τ*_eff_/*τ*_0_ as a function of CRP concentration at 300 K, expressed on a semilog plot, where Δ*τ*_eff_ = [*τ*_eff_ (*t* = 0) − *τ*_eff_ (*t* = 6000 s)] when the association of CRP with Fe_3_O_4_-antiCRP is complete. The effective relaxation time *τ*_0_ = 0.62 μs at t = 0 is derived from tan*θ* = *ωτ*_eff_. When the concentration of CRP is 20 ppm, Δ*τ*_eff_ = 0.26 μs (Δ*τ*_eff_/*τ*_0_ = 41.9%) and, when the CRP concentration decreases to 0.1 ppm, Δ*τ*_eff_ saturates at 0.11 μs (Δ*τ*_eff_/*τ*_0_ = 17.7%). If we further reduce the CRP concentration to 0.01 ppm, Δ*τ*_eff_ remains at ~0.11 μs, which is close to the experimental noise. Thus, for the present system, the lowest concentration for which detection is possible is 0.1 ppm.

The ratio Δ*τ*_eff_/*τ*_0_ as a function of CRP concentration follows a characteristic logistic function [15] given by

$$\Delta \tau_{\text{eff}}/\tau_{\text{0}}=\frac{A-B}{1+{\left(\frac{\phi_{\text{CRP}}}{\phi_{0}}\right)}^{\beta }}+B$$ (4)

where *A* and *B* are dimensionless parameters, and *ϕ*_CRP_ and *ϕ*_0_ are in the units of ppm (or μg/mL). The solid line in Fig 4 shows a fit with parameters *A* = 17.3, *B* = 41.0, *β* = 3.79, and *ϕ*_0_ = 3.60 ppm. Because of environmental and system noise, Δ*τ*_eff_/*τ*_0_ is nonzero at *ϕ*_CRP_ = 0. In practical applications, this universal logistic function provides a basis for estimating the quantity of biomolecules [16].

From Eq 2 we obtain the magnitude of the ac susceptibility, *χ*_amp_, which is given by

$$\chi_{\text{amp}}(t)=\frac{\chi_{0}}{{\left[1+{\left(\omega \tau_{\text{eff}}(t)\right)}^{2}\right]}^{1/2}}.$$ (5)

**Fig 5:**
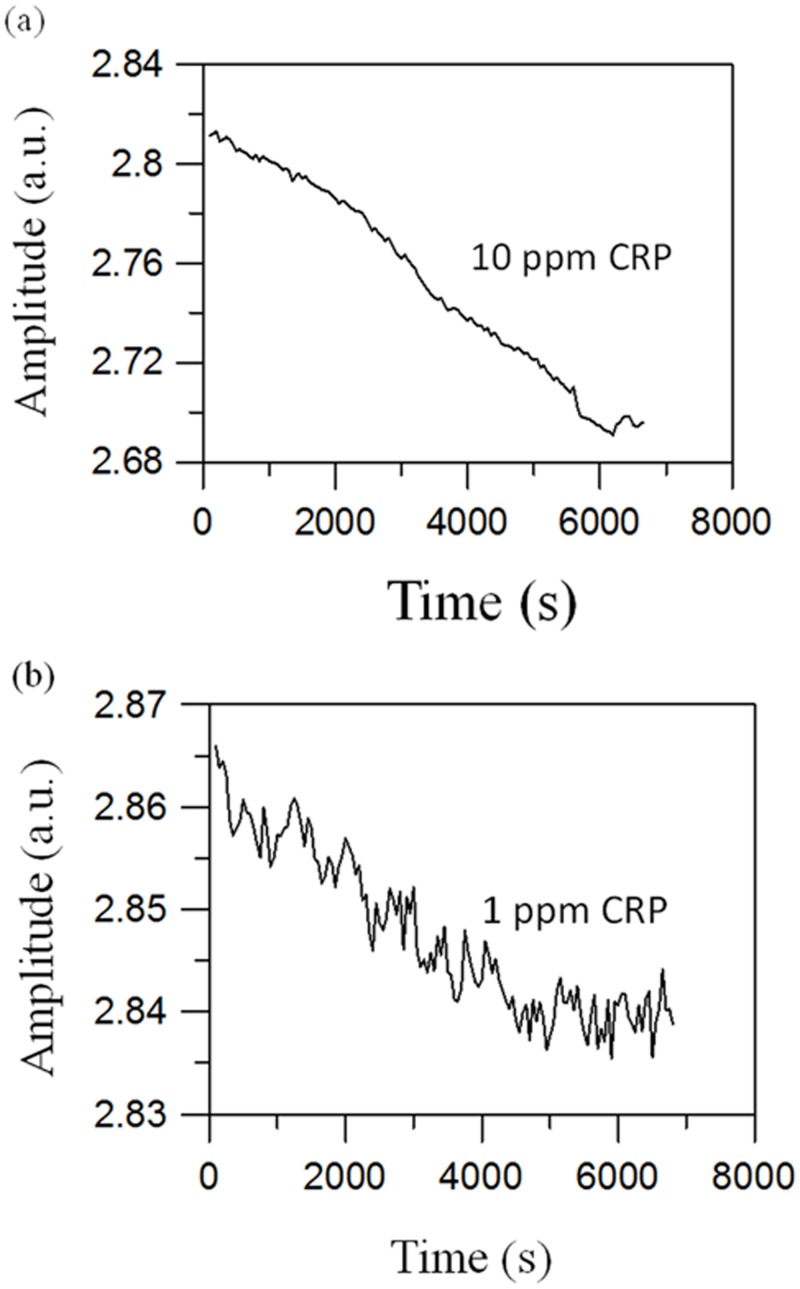
Representative amplitude of ac susceptibility as a function of time when assaying (a) 10 ppm CRP and (b) 1.0 ppm CRP. The increase in *τ*_eff_ induces a reduction of *χ*_amp_ during particle association.

Based on Eq 5 we see that an increase in *τ*_eff_ induces a reduction of *χ*_amp_ after association. Additionally, assaying a higher concentration of CRP leads to a larger reduction in Δ*χ*, which is observed in experiments. Fig 5 shows the time-dependent amplitude of the ac susceptibility for assaying 10 and 1.0 ppm CRP. The susceptibility *χ*(*t*) decreases from 2.83 μV at t = 0 to 2.70 μV at t = 6000 s when assaying 10 ppm CRP, and from 2.87 μV at t = 0 to 2.84 μV at t = 6000 s when assaying 1 ppm CRP. The percent reduction Δ*χ*/*χ*_0_ × 100% = 4.6% when assaying 10 ppm CRP, and Δ*χ*/*χ*_0_ was reduced to 0.8% when assaying 1.0 ppm CRP.

**Fig 6:**
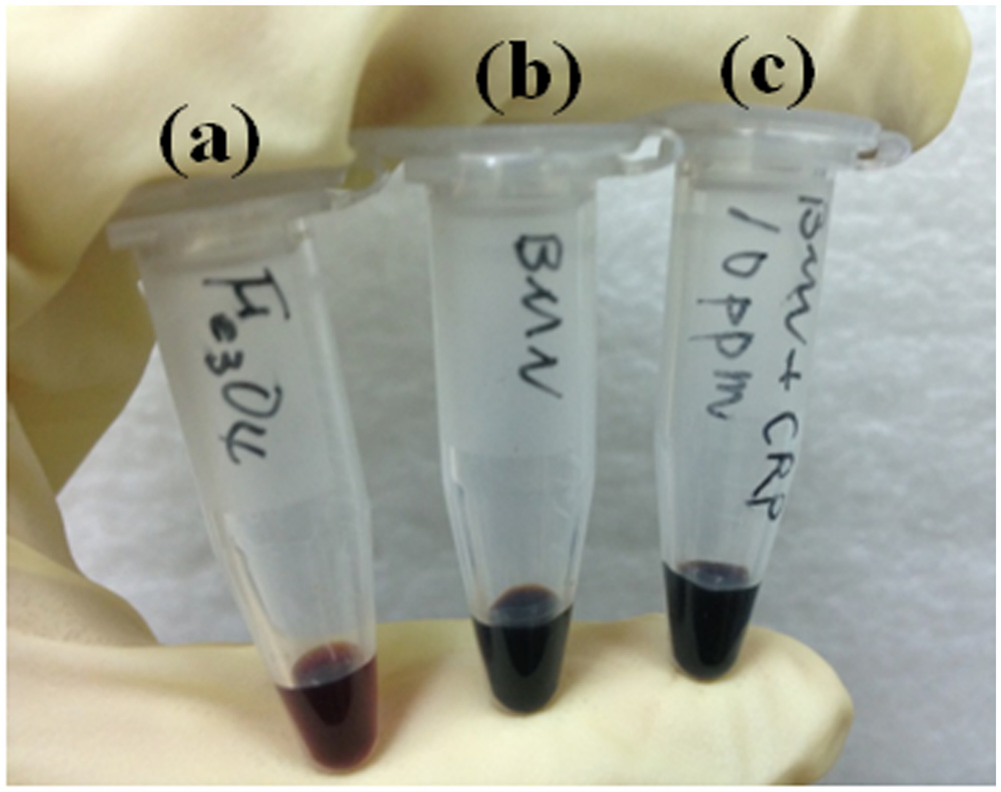
The picture of the solutions of Fe_3_O_4_ nanoparticles, Fe_3_O_4_-antiCRP, and CRP conjugated Fe_3_O_4_-antiCRP after 1.5 hours mixture. (a) Fe_3_O_4_ 60μl + PBS 40μl, (b) Fe_3_O_4_-antiCRP 60μl + PBS 40μl, and (c) Fe_3_O_4_-antiCRP 60μl + 10 ppm CRP antigen 40μl after 1.5 hours.

**Fig 7:**
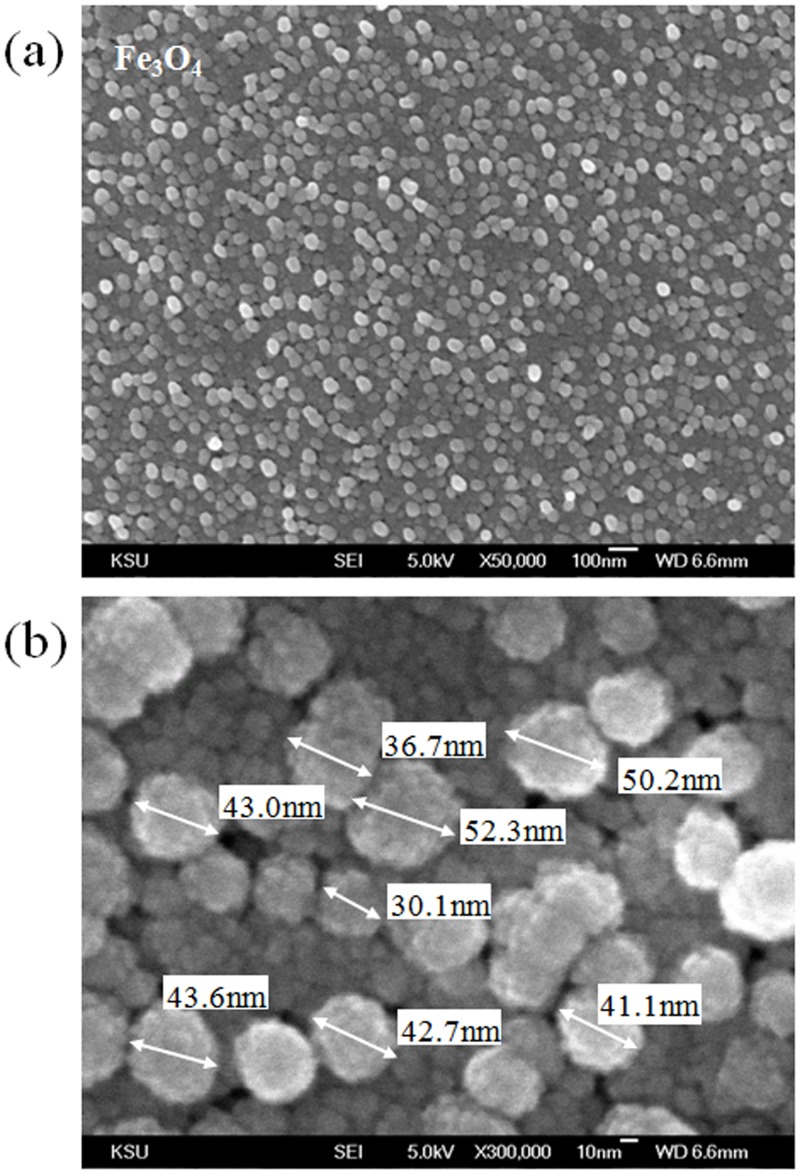
SEM images of (a) Fe_3_O_4_ applied to BMN synthesis. (b) Magnified SEM picture of Fe_3_O_4_ with the particle size indicated. The average size of Fe_3_O_4_ particles is ~ 48.4 ± 9.9 nm.

Although the measurement of ac susceptibility provides evidence for magnetic clustering from the conjugation of CRP and Fe_3_O_4_-antiCRP, the aggregation is not directly observed. Fig 6 is the picture of the solutions of Fe_3_O_4_ nanoparticles, Fe_3_O_4_-antiCRP, and CRP conjugated Fe_3_O_4_-antiCRP after 1.5 hours mixture. It clearly shows that there are not any observable aggregations and precipitations in these solutions even though CRP had conjugated with Fe_3_O_4_-antiCRP for 1.5 hours. Therefore, we used the SEM and TEM to investigate the effect of clustering. The inhomogeneity of BMNs usually leads to large variations in results. Thus, to enhance the stability and reproducibility of assaying biomarkers, highly homogeneous Fe_3_O_4_ is necessary for the Fe_3_O_4_-antiCRP preparation. We used SEM to check the homogeneity of the Fe_3_O_4_ use for BMN synthesis. Fig 7a shows a typical SEM image of Fe_3_O_4_, and Fig 7b shows a magnified SEM image of Fe_3_O_4_, with the particle size indicated. The diameters of the Fe_3_O_4_ are almost all the same, which indicates that the Fe_3_O_4_ used in the BMN synthesis is highly homogeneous. The particle size of Fe_3_O_4_ was measured to be ~ 48.4 ± 9.9 nm on average, which is very close to the result obtained with dynamic light scattering.

**Fig 8:**
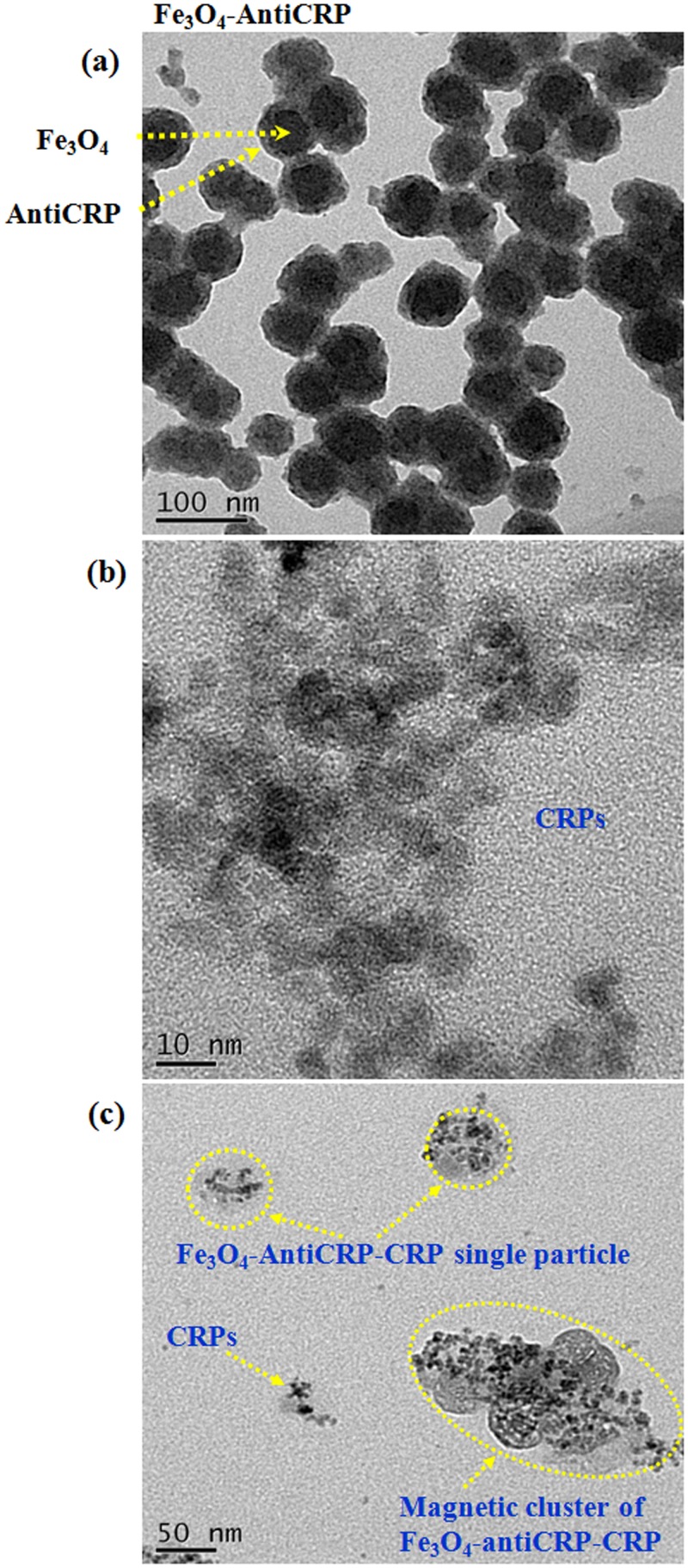
TEM images of (a) Fe_3_O_4_-antiCRP nanoparticles and (b) CRP antigens. (c) TEM image of conjugated Fe_3_O_4_-antiCRP-CRP particles and cluster after association time of 1 h.

Fig 8a shows a TEM image of the reagent composed of Fe_3_O_4_-antiCRP. The average particle size varies from (60 ± 5.0) to (70 ± 5.0) nm with an average size of ~ 67.5 ± 11.5 nm. The core of Fe_3_O_4_ and the outer shell of the coated antiCRP are apparent. Moreover, Fig 8b shows a TEM image of CRP. The average particle size of CRP is 10 ± 0.5 nm which is consistent with the data reported in Ref. [17]. TEM shows that CRP conjugates with Fe_3_O_4_-antiCRP upon adding CRP to the reagent of Fe_3_O_4_-antiCRP. Fig 8c shows a TEM image of the magnetic cluster of Fe_3_O_4_-antiCRP-CRP at *t* = 1 h after mixing CRP with Fe_3_O_4_-antiCRP. In addition to the unconjugated CRPs, the conjugated Fe_3_O_4_-antiCRP-CRP single particles and the Fe_3_O_4_-antiCRP-CRP cluster are also apparent. These results show clearly that CRP adheres to the surface of Fe_3_O_4_-antiCRP via antigen-antibody interactions [18] and aggregates Fe_3_O_4_-antiCRP together to form bigger clusters. The size of magnetic clusters of Fe_3_O_4_-antiCRP-CRP varies from ~ 80 to 250 nm at t = 1 h and increases with association time. Thus, the effect of magnetic clustering is directly revealed by SEM and TEM.

A method to conduct a wash-free magnetically labeled immunoassay using mixed excitation frequencies was previously proposed [4]. As a detection method, two excitation currents at frequencies of *f*_1_ and *f*_2_ were applied to the excitation coils. The reduction of *χ*_ac_ for BMNs conjugated with biotargets was analyzed at the target frequency *f*_1_+*2f*_2_ to qualitatively determine the amount of biotarget, where *f*_1_ and *f*_2_ are the excitation frequencies of the input coils. The IMR assay in the frequency domain was used to assay CRP [19,20]. Real-time Brownian relaxation of BMNs was also investigated by using a mixed frequency method, and the phase delay between *M*(*t*) and *H*(*t*) was investigated to detect biomolecules [9]. In the present work, the phase delay of *M* with respect to *H* and the reduction of *χ*_amp_ was investigated to assay CRP by using single frequency ac susceptometry. The results indicate that *τ*_eff_ increases with association time. We attribute this result to the suppression of Brownian motion, which is due to the magnetic clustering during association. Additionally, the ratio Δ*τ*_eff_/*τ*_0_ follows a logistic function, which provides a basis for estimating the quantity of biomolecules. Furthermore, the effect of magnetic clustering is directly revealed via SEM and TEM.

CRP levels in blood are key indicators of infectious disease, noninfectious disease, and acute tissue. The normal concentration in healthy human serum is usually less than 10 μg/mL, which slightly increases with age. Higher levels are found in late-pregnancy women and in people with mild inflammation or viral infection (10–40 μg/mL), in people with active inflammation or bacterial infection (40–200 μg/mL), and in people with severe bacterial infections or burns (>200 μg/mL) [21]. By using BMNs and the technique of mixed-frequency ac susceptmetry, quantitative immunoassays for CRP were demonstrated in clinical diagnoses [22]. In the present work, we investigate the dynamics of *τ*_eff_ and use SEM and TEM to show magnetic clustering. By using *θ*(*t*) and *τ*_eff_(*t*), this method provides a detection sensitivity of 0.1 ppm.

## Conclusions

We investigate the dynamics of *τ*_eff_(*t*) during association of Fe_3_O_4_-antiCRP with CRP. The results show that *τ*_eff_ increases and saturates when association is complete. In addition, the ratio Δ*τ*_eff_/*τ*_0_ as a function of CRP concentration follows a characteristic logistic function, which provides a basis for estimating the quantity of biomolecules and a detection sensitivity of 0.1 ppm is demonstrated. Finally, SEM and TEM images directly revealed the formation of magnetic clusters. This magnetic detection platform is promising for future use in assaying a broad number of biomarkers, such as viruses, proteins, tumors, etc.
